# Nuclear basic fibroblast growth factor regulates triple-negative breast cancer chemo-resistance

**DOI:** 10.1186/s13058-015-0590-3

**Published:** 2015-07-04

**Authors:** Shenduo Li, Sturgis Payne, Fang Wang, Peter Claus, Zuowei Su, Jeffrey Groth, Joseph Geradts, Gustaaf de Ridder, Rebeca Alvarez, Paul Kelly Marcom, Salvatore V. Pizzo, Robin E. Bachelder

**Affiliations:** Department of Pathology, Duke University Medical Center, P.O. Box 3712, Durham, N.C. 27710 USA; Duke Cancer Institute, Durham, N.C. 27710 USA; Institute of Neuroanatomy, Hannover Medical School, Hannover, Germany

## Abstract

**Introduction:**

Chemotherapy remains the only available treatment for triple-negative (TN) breast cancer, and most patients exhibit an incomplete pathologic response. Half of patients exhibiting an incomplete pathologic response die within five years of treatment due to chemo-resistant, recurrent tumor growth. Defining molecules responsible for TN breast cancer chemo-resistance is crucial for developing effective combination therapies blocking tumor recurrence. Historically, chemo-resistance studies have relied on long-term chemotherapy selection models that drive genetic mutations conferring cell survival. Other models suggest that tumors are heterogeneous, being composed of both chemo-sensitive and chemo-resistant tumor cell populations. We previously described a short-term chemotherapy treatment model that enriches for chemo-residual TN tumor cells. In the current work, we use this enrichment strategy to identify a novel determinant of TN breast cancer chemotherapy resistance [a nuclear isoform of basic fibroblast growth factor (bFGF)].

**Methods:**

Studies are conducted using our in vitro model of chemotherapy resistance. Short-term chemotherapy treatment enriches for a chemo-residual TN subpopulation that over time resumes proliferation. By western blotting and real-time polymerase chain reaction, we show that this chemotherapy-enriched tumor cell subpopulation expresses nuclear bFGF. The importance of bFGF for survival of these chemo-residual cells is interrogated using short hairpin knockdown strategies. DNA repair capability is assessed by comet assay. Immunohistochemistry (IHC) is used to determine nuclear bFGF expression in TN breast cancer cases pre- and post- neoadjuvant chemotherapy.

**Results:**

TN tumor cells surviving short-term chemotherapy treatment express increased nuclear bFGF. bFGF knockdown reduces the number of chemo-residual TN tumor cells. Adding back a nuclear bFGF construct to bFGF knockdown cells restores their chemo-resistance. Nuclear bFGF-mediated chemo-resistance is associated with increased DNA-dependent protein kinase (DNA-PK) expression and accelerated DNA repair. In fifty-six percent of matched TN breast cancer cases, percent nuclear bFGF-positive tumor cells either increases or remains the same post- neoadjuvant chemotherapy treatment (compared to pre-treatment). These data indicate that in a subset of TN breast cancers, chemotherapy enriches for nuclear bFGF-expressing tumor cells.

**Conclusion:**

These studies identify nuclear bFGF as a protein in a subset of TN breast cancers that likely contributes to drug resistance following standard chemotherapy treatment.

## Introduction

Targeted therapies are not available for triple-negative (TN) breast cancer, which lacks estrogen receptor, progesterone receptor, and human epidermal growth factor receptor-2 (HER2) over-expression. Although TN breast tumors initially respond to chemotherapy, this response is incomplete in more than half of these patients [1, 2]. Notably, tumor recurrence is observed within 5 years of treatment in half of patients exhibiting an incomplete pathologic response, resulting in patient mortality [3, 4]. Accumulating evidence indicates that a small population of drug-resistant tumor cells surviving initial chemotherapy treatment is likely responsible for tumor relapse [5–7]. In order to identify new treatment strategies for these aggressive breast cancers, there is an urgent need to identify novel signaling pathways that contribute to TN breast cancer chemo-resistance.

We previously characterized an in vitro model of chemo-resistance/tumor recurrence [8]. In this model, tumor cells were subjected to short-term chemotherapy, which killed 99.9 % of tumor cells. However, a subpopulation (0.1 %) of chemo-resistant tumor cells persisted and resumed proliferation approximately 2 weeks after chemotherapy removal. In the current work, we investigated signaling pathways that drive TN tumor cell chemo-resistance using this in vitro model.

The basic fibroblast growth factor family (bFGF) (alternatively known as FGF-2) consists of both cytosolic (secreted) and nuclear isoforms. Expression of these bFGF isoforms is regulated at the level of translation. Specifically, cytosolic forms (low molecular weight, 18 kDa) are regulated by cap-dependent translation, whereas nuclear forms (high molecular weight; 22, 22.5, and 24 kDa) are regulated by cap-independent translation [9]. These isoforms differ in molecular weight because they utilize different translation initiation sites.

Cytosolic (secreted) isoforms of bFGF are implicated in tumor resistance to anti-angiogenic therapy [10–15]. However, functions for nuclear bFGF in cancer cells remain poorly understood. In over-expression models, nuclear bFGF has been reported to regulate cell cycle [16–18], cell survival [19], radio-resistance [20], and tumor metastasis [19, 21]. Moreover, nuclear bFGF expression in astrocytic tumors is associated with a poor patient prognosis [22]. To date, nuclear bFGF expression/function in breast cancer has not been investigated.

DNA repair pathways are frequently de-regulated in breast cancer. Whereas BRCA proteins are responsible for homologous repair, DNA-dependent protein kinase (DNA-PK) repairs double-stranded DNA breaks by non-homologous end joining. DNA-PK consists of a catalytic subunit (DNA-PK_CS_) and a regulatory subunit (Ku70 and Ku80 heterodimer), which recruits DNA-PK_CS_ to DNA. The status of the cell cycle determines whether DNA-PK or BRCA repairs DNA, with DNA-PK being responsible in growth-arrested cells [23].

Previous studies using bFGF over-expression models suggest that nuclear bFGF drives DNA-PK_CS_ transcription [20]; however, the ability of endogenous bFGF to regulate DNA-PK_CS_ expression/DNA repair in tumor cells has not been reported. In the current work, we show that nuclear bFGF promotes survival of chemo-residual TN tumor cells. This bFGF function is associated with increased DNA damage repair mediated by increased DNA-PK expression/activity. Our work identifies nuclear bFGF as a central determinant of TN breast cancer chemo-resistance, and suggests a novel therapeutic target (nuclear bFGF) for preventing TN breast cancer recurrence.

## Methods

### Cell culture

SUM159 TN breast cancer cells were obtained from the Duke Cell Culture Facility and maintained in Ham’s F-12 medium containing 5 % heat-inactivated FBS, 5 μg/ml insulin, and 1 μg/ml hydrocortisone. BT549 TN breast cancer cells were obtained from the Duke Cell Culture Facility and maintained in RPMI 1640 medium containing 10 % heat-inactivated FBS, 1 μg/ml insulin, 10 mM HEPES, 1 mM pyruvate, and 2.5 g/L glucose.

### Generation of chemo-residual tumor cells and subsequent colonies

SUM159 tumor cells were seeded in T225 cell culture flasks (2 × 10^6^ cells/flask) and, after 2 days, treated with either 1 μg/ml doxorubicin (Sigma, St Louis, MO, USA) or 100 nM docetaxel (Sigma). The drug was removed after 2 days, and cells were fed new media every third day. The majority of cells (99.9 %) were eliminated by day 7, after which only chemo-residual cells (0.1 %) were observed. BT549 tumor cells were seeded in T225 cell culture flasks (3 × 10^6^ cells/flask) and, after 2 days, treated with 0.5 μg/ml doxorubicin. Drug was removed after 2 days, and cells were fed new media every third day. The majority of cells (99.9 %) were eliminated by day 7, after which only chemo-residual cells (0.1 %) were observed. SUM159 and BT549 chemo-residual cells were harvested on day 7 with trypsin-EDTA, and re-plated in 6-well plates. Resumed proliferation of chemo-residual tumor cells was monitored over time. Medium was changed every 3 to 4 days. Colonies evolving from chemo-residual cells were stained with crystal violet and colonies containing >50 cells were counted [24].

For the mammosphere culture, cells were seeded into Mammacult media (Stem cell Tech., Vancouver, BC, Canada; #05620) supplemented with 1 % Methylcellulose (Sigma St Louis, MO, USA; #M0430), penicillin/streptomycin (Life Technologies, Carlsbad, CA, USA), heparin (Stemcell Tech., #07980; 4 μg/mL), and hydrocortisone (1 μg/ml). The sphere assay was setup in Costar 6-Well Ultra Low Attachment (Corning, NY, USA) plates in triplicate. Cells were incubated at 37 °C/5 % CO2. Spheres were counted with a GelCount cell counter (Oxford Optronix, Milton Park, UK) after 3 to 7 days. For secondary sphere assays, primary spheres were dissociated with trypsin, washed, and seeded at 20,000 cells/well as above.

### Western blots

Cells were harvested using trypsin-EDTA and washed with PBS. Harvested cells were incubated in cytosolic lysis buffer (10 mM HEPES (pH 7.4), 10 mM KCl, 1.5 mM MgCl_2_, 0.5 % NP40, and proteinase inhibitors) on ice for 20 minutes and centrifuged. Supernatants were collected as cytosolic protein lysates. The residual pellets were washed with cytosolic lysis buffer, and then incubated in nuclear lysis buffer (50 mM TRIS (pH 7.5), 1 % SDS, and proteinase inhibitors) plus Benzonase (Sigma) on ice for 20 minutes. The supernatants after centrifugation were collected as nuclear protein extracts. Protein concentrations were determined by bicinchoninic acid (BCA) assay. Equivalent amounts of protein were subjected to SDS-polyacrylamide gel electrophoresis (PAGE) and immunoblotted with the following primary antibodies, followed by the appropriate species IRDye-conjugated secondary antibody (Life Technologies, Carlsbad, CA, USA): bFGF (BD Biosciences, Franklin Lakes, NJ, USA; catalog # 610073), Lamin-A (Sigma), DNA-PK_CS_ (Cell Signaling, Beverly, MA, USA; catalog # 4602), phospho-Ser 2056-DNA-PK_CS_ (Cell Signaling, Beverly, MA, USA; catalog # 4215), GAPDH (GenScript, Piscataway, NJ, USA). Proteins were detected using Odyssey infrared imaging system (LI-COR, Lincoln, NE, USA).

### Immunofluorescence

Cells were grown on glass coverslips, washed with Hanks’ balanced salt solution (HBSS), and fixed for 30 minutes at room temperature in 1 % fresh formaldehyde in PBS. After washing for 5 minutes in PBS, the coverslips were incubated in 5 % bovine serum albumin (BSA) in PBS for 90 minutes at room temperature. Excess BSA was drained from the coverslips, and cells were incubated with the primary bFGF antibody (BD Biosciences, Franklin Lakes, NJ, USA) in PBS containing 0.5 % BSA, overnight at 4 °C. The cells were then rinsed three times in PBS containing 0.1 % Tween and incubated with secondary antibody (1:400 dilution of an Alexa Fluor 568-conjugated donkey anti-mouse IgG, Life Technologies) for 90 minutes at 4 °C in the dark. The cells were washed three times with PBS, incubated with a 1:1000 dilution of Hoechst (Life Technologies) for 10 minutes at room temperature in the dark, and washed three times with PBS. The coverslips were dried for 2 h at room temperature, mounted, and cured overnight at 4 °C. Pictures were taken using a fluorescence microscope and analyzed with Gen5 image analysis software (BioTek, Winooski, VT, USA).

### Thymidine uptake

Cells were plated in 96-well plates (3 × 10^3^ cells/well). After 4 h, cells were incubated with 0.5 μCi/well Methyl-[^3^H]-Thymidine (Perkin Elmer, Waltham, MA, USA) for 16 h before harvesting onto glass-fiber filters. [^3^H]-Thymidine incorporation was measured as counts per minute (CPM) using a Tri-Carb 2100TR time-resolved liquid scintillation counter (Perkin Elmer, Waltham, MA, USA).

### Alamar blue

Cells were plated in 96-well black, clear-bottom plates (2 × 10^3^ cells/well) in 100 μl complete medium. After 4 h, 10 μl/well Alamar Blue (Life Technologies) reagent was added. After 2 h, fluorescence was measured using a Cytation3 plate reader (BioTek).

### shRNA and addback transfection

Cells were grown to 50 % confluence in a 10-cm dish. The transfection mixtures contained: 1) 2 μg bFGF shRNA (Sigma, St. Louis, MO; clone # NM_02006.x-635s1c1; catalog # TRCN0000003330) or control shRNA plasmid (Sigma, St Louis, MO, USA; pLKO.1) + Optimem (Life Technologies) and 2) Lipofectamine 2000 (Life Technologies) + Optimem. These mixtures were incubated separately at room temperature for 5 minutes, combined, and incubated for 30 minutes at room temperature. Cells were washed twice with HBSS (Life Technologies). Optimem was then added to the RNA/Lipofectamine mixture, and the mix was added to the cells, which were incubated overnight at 37 °C. This medium was removed the next day and replaced with media containing puromycin (5 μg/ml, SUM159; 2 μg/ml, BT549). Cells were expanded in puromycin and tested for bFGF knockdown by western blotting. For bFGF addback, plasmids [25] expressing 18-kDa rat bFGF, 23-kDa rat bFGF, or an empty control vector were transfected into SUM159 or BT549 cells stably expressing a bFGF shRNA. The transfection protocol was performed as above, except that the cells were selected in puromycin (as above) and G418 (Life Technologies) at 400 μg/ml. Expression of addback constructs was assessed by western blotting cell extracts with bFGF antibody.

### Single cell gel electrophoresis assay (comet assay)

Cells were challenged with doxorubicin (SUM159: 1 μg/ml, 3 h; BT549: 0.5 μg/ml, 4h). Fresh medium was added after chemotherapy removal. Cells were harvested at sequential time points after chemotherapy, mixed with low-melting-point agarose, and spread on comet slides using a Trevigen CometAssay® Kit (Trevigen Inc., Gaithersburg, MD, USA). After incubation with lysis solution and neutral solution, slides were subjected to electrophoresis at 19 V for 50 minutes under neutral conditions. Slides were incubated with DNA precipitation solution (1 M NH_4_A_C_, 95 % EtOH) for 30 minutes, followed by 70 % ethanol for 30 minutes. Slides were then stained with a 1:500 dilution of Hoechst (Life Technologies) for 15 minutes and washed with PBS. Samples were examined using a fluorescence microscope, and the presence of comet tails was quantified using Gen5 image analysis software (BioTek). Cells from three fields were analyzed for each time point. Each field contained at least 50 cells.

### Real-time quantitative RT-PCR

Total RNA from SUM159 cells was extracted using PrepEase® RNA Spin Kit (USB, Cleveland, OH, USA) and treated with RNase-free DNase to remove residual genomic DNA. Single-stranded cDNAs were synthesized using the iScript cDNA synthesis kit (Bio-Rad, Hercules, CA, USA). Human fibroblast growth factor-2 (FGF2) and human DNA-PKcs primers were purchased from realtimeprimers.com (Elkins Park, PA, USA). Real-time PCR on the Mx3005P® QPCR System (Stratagene, La Jolla, CA, USA) was performed in the presence of 12.5 μl VeriQuest™ Fast SYBR Green qPCR Master mix (2×) (USB, Cleveland, OH), 2 μl cDNA, and H_2_O added to a final volume of 25μl. The mixtures were denatured for 5 minutes at 95 °C, followed by 40 cycles of 3 s at 95 °C, and 30 s at an annealing temperature at 60 °C. PCR products were monitored in real time by measuring the increase in fluorescence caused by the binding of SYBR Green I Dye. Significance was analyzed using the software package MxPro™ QPCR Software (Stratagene, La Jolla, CA, USA).

### Selective DNA-PK inhibitor (NU7441) studies

Cells were seeded in T225 cell culture flasks (2 × 10^6^ cells/flask) and, after 2 days, treated with 1 μg/ml doxorubicin (Sigma) plus dimethyl sulfoxide (DMSO) or NU7441 (1 μM or 5 μM, R&D Systems, Minneapolis, MN, USA). The drug was removed after 2 days, and cells were fed with new medium every third day. Chemo-residual cells were harvested on day 7 with trypsin-EDTA, and re-plated in 6-well plates. Medium was changed every 3 to 4 days. Colonies evolving from chemo-residual cells were stained with crystal violet. Colonies containing >50 cells were counted.

### Immunohistochemistry of patient tumors

TN breast cancer patients treated with neoadjuvant chemotherapy that exhibited an incomplete pathologic response were identified from medical records under Duke Institutional Review Board approval (protocol 47289). Retrospectively collected tumor biopsies (obtained pre-chemotherapy) and biopsies/resections (obtained post-chemotherapy) from these patients were retrieved. Formalin-fixed, paraffin-embedded tissues were subjected to bFGF immunohistochemistry. Slides were baked at 60 °C for 1 h, and deparaffinized in xylene followed by 100 % ethanol. Antigen retrieval was performed in sub-boiling EDTA (pH 8) at 100 °C for 40 minutes. bFGF staining was performed in an autostainer according to the following program: Endogenous Peroxidase Quench (Cell Marque Rocklin, CA, USA) 5 minutes; Protein block (Biocare Medical, Concord, CA, USA), 10 minutes; bFGF antibody (BD Biosciences, Franklin Lakes, NJ, USA; catalog # 610073 (1:250 dilution), 1 h; secondary detection kit (Cell Marque, Rocklin, CA, USA), 20 minutes; 3,3-diaminobenzidine (DAB), 5 minutes; hematoxylin, 1 minute; and Bluing, 1 minute. Slides were then placed in water and dehydrated in xylene before adding a coverslip.

### bFGF immunohistochemistry scoring

Two pathologists (GD, RA) (blinded to patient samples) assigned scores for the percentage of nuclear bFGF-positive cells and percentage of cytosolic bFGF-positive cells. The scoring of all cases was confirmed by a board-certified pathologist (SVP).

## Results

### Chemo-residual TN breast cancer cells express increased nuclear bFGF

**Fig. 1:**
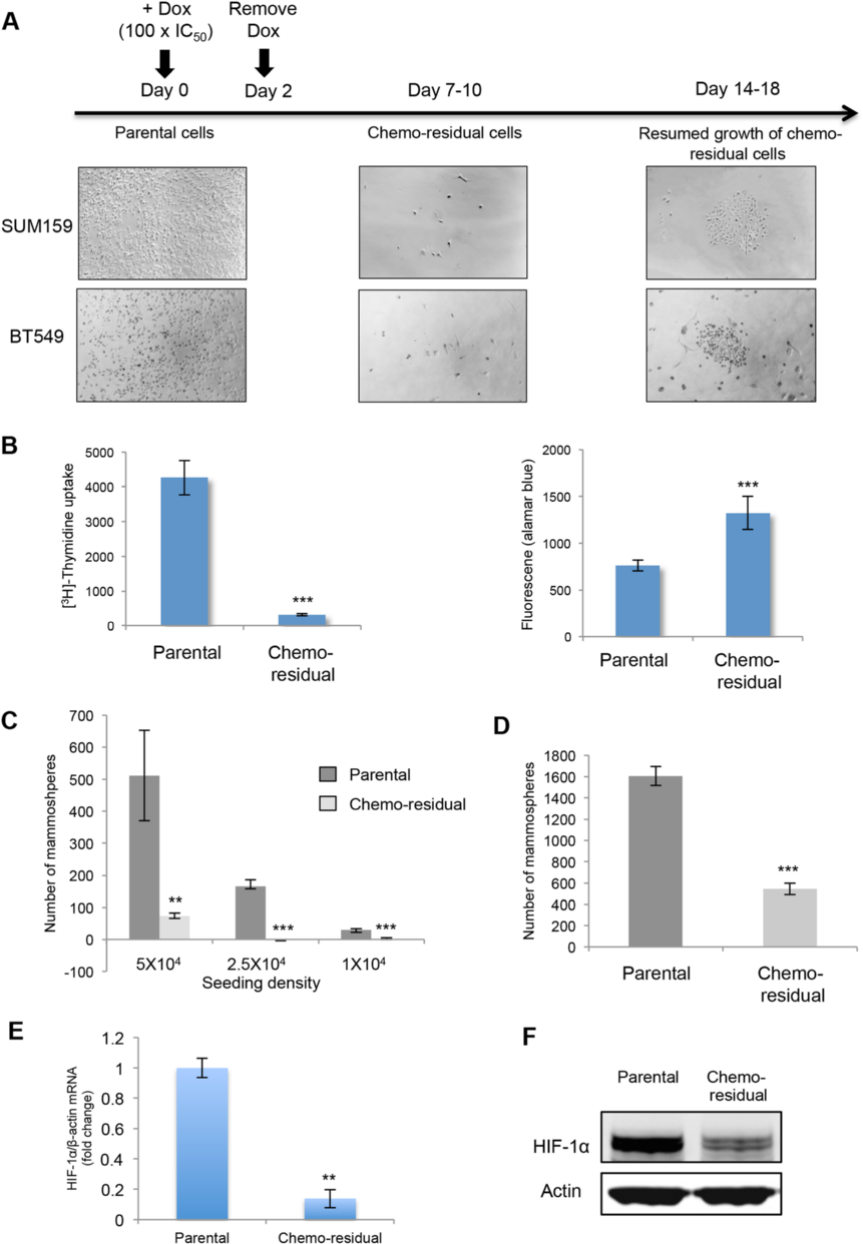
Chemo-residual triple-negative (*TN*) breast tumor cells emanating from a short-term chemotherapy treatment model do not exhibit cancer stem-like cell behavior. **a** In vitro model of TN breast cancer chemo-resistance. SUM159 and BT549 tumor cells were treated with doxorubicin (*Dox*) (1 μg/ml, 0.5 μg/ml, respectively) for 2 days, after which chemotherapy was removed and fresh medium was added. Between 7 and 10 days, a small number of chemo-residual cells (0.1 % of the original population) remained, and exhibited reduced proliferation compared to parental (untreated) cells. Approximately 2 weeks after chemotherapy withdrawal, chemo-residual cells resumed proliferation and established colonies. Pictures of parental (untreated) cells, chemo-residual cells, and colonies emanating from chemo-residual cells were taken on days 0, 7, 14 (SUM159 cells) or days 0, 10, 18 (BT549 cells), respectively. Magnification ×200. Similar data were obtained by treating SUM159 cells with docetaxel (100 nM) for 2 days [8]. **b** SUM159 cells were treated with *Dox* as described in Fig. 1a. Parental and chemo-residual cells were seeded at equal density in a 96-well plate. *Left panel*: proliferation was determined by ^3^H-thymidine incorporation. *Right panel*: cell viability was assessed by alamar blue. *Error bars* represent SD, n = 6, ***p <0.001, two-tailed Student’s *t* test. **c** SUM159 parental and chemo-residual cells harvested on day 7 after *Dox* treatment were seeded at the indicated numbers into a mammosphere assay. Mammosphere number was quantified after 4 days. *Error bars* represent SD, n = 3, **p <0.01, ***p <0.001, two-tailed Student’s *t* test. **d** Parental and chemo-residual cells harvested on day 8 after docetaxel treatment were seeded into a mammmosphere assay. Mammosphere number was quantified after 4 days. *Error bars* represent SD, n = 3, ***p <0.001, two-tailed Student’s *t* test. **e** RNA was extracted from SUM159 parental and chemo-residual cells harvested on day 7 after *Dox* treatment. Equivalent amounts were subjected to hypoxia inducible factor-1α (*HIF-1α*) real-time PCR. Results are expressed as the mean *HIF-1α*/β- actin ratio from triplicate wells (+/- SD). **p <0.01, two-tailed Student’s *t* test. **f** Total cellular extracts obtained from SUM159 parental and chemo-residual cells harvested on day 7 after *Dox* treatment were subjected to SDS-PAGE and immunoblotted with a *HIF-1α* or β-actin antibody, followed by secondary antibody

To enrich for chemo-resistant tumor cells, we treated SUM159 and BT549 TN breast tumor cells with doxorubicin at a clinically relevant concentration [26]. Doxorubicin was removed after 2 days, and fresh medium was added. Although the vast majority of cells were eliminated by day 6, we observed a small number of residual, viable tumor cells, representing 0.1 % of the original population, on day 7 post treatment (Fig. 1a). These chemo-residual cells were metabolically active, but exhibited significantly reduced proliferation (Fig. 1b). Chemo-residual tumor cells resumed proliferation approximately 2 weeks post chemotherapy treatment. Our previous studies show that colonies from this model continue to expand after 18 days, and exhibit resistance to multiple classes of chemotherapy for as long as 50 days [8].

Previous studies suggest that continuous chemotherapy selection models promote the growth of cancer stem-like cells [27–29]. Accordingly, we investigated if chemo-residual tumor cells from our short-term chemotherapy treatment model behaved like cancer stem-like cells. As shown in Fig. 1c, d, chemo-residual TN tumor cells in our model did not exhibit increased mammosphere-forming ability. Continuous chemotherapy treatment of TN breast cancer cells has also been shown to drive hypoxia inducible factor-1α (HIF-1α) expression, a critical determinant of cancer stemness [28]. In contrast, chemo-residual tumor cells emanating from our short-term chemotherapy treatment model exhibited reduced HIF-1α mRNA (Fig. 1e) and protein (Fig. 1f) levels. Collectively, these findings suggest that chemo-residual tumor cells evolving from our short-term chemotherapy treatment model are different from previously defined cancer stem-like cells selected by continuous chemotherapy treatment models.

**Fig. 2:**
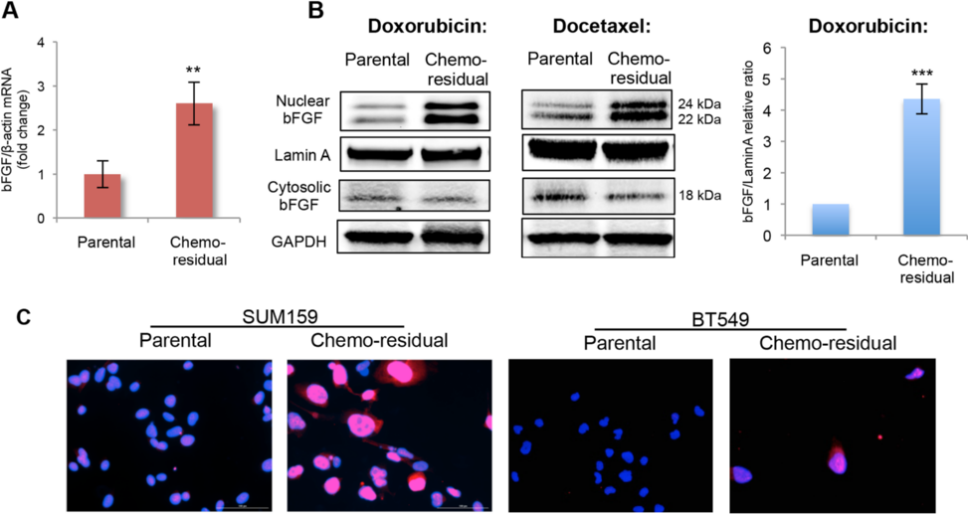
Chemo-residual triple-negative (*TN*) breast cancer cells from a short-term chemotherapy treatment model exhibit increased expression of a nuclear isoform of basic fibroblast growth factor (*bFGF*). **a** Increased bFGF RNA in chemo-residual SUM159 cells after doxorubicin treatment as described in Fig. 1a. Total RNA was extracted from parental and chemo-residual cells. bFGF mRNA expression was quantified by qRT-PCR, and is shown as fold increase relative to β-actin. *Error bars* represent SD, n = 3, **p <0.01, two-tailed Student’s *t* test. **b** *Left panel*: increased expression of nuclear, but not cytosolic bFGF in chemo-residual SUM159 cells after doxorubicin or docetaxel treatment (as described in Fig. 1a). Nuclear or cytosolic protein was extracted from parental and chemo-residual cells. Equivalent amounts were immunoblotted with bFGF, Lamin A, or GAPDH antibody, followed by IrDye-conjugated secondary antibodies. Protein bands were detected by infrared imaging. *Right panel*: protein bands from three independent trials (doxorubicin treatment, as described in Fig. 1a) were quantified using Image J software (NIH), and the relative ratio of nuclear bFGF to loading control is shown for parental and chemo-residual SUM159 cells. *Error bars* represent SD, n = 3, ***p <0.001, two-tailed Student’s *t* test. **c** SUM159 and BT549 cells were treated with doxorubicin as described in Fig. 1a. Parental and chemo-residual cells were fixed and stained with Hoechst (*blue*) and bFGF antibody (*red*) to demonstrate the increased nuclear localization of bFGF in chemo-residual TN breast cancer cells. Magnification ×400.

bFGF signaling has been implicated in tumor resistance to targeted therapies [10–15]. Accordingly, we investigated bFGF expression in chemo-residual TN tumor cells from our short-term chemotherapy treatment model. As shown in Fig. 2a, we observed significantly increased bFGF mRNA expression in chemo-residual tumor cells compared to parental cells. To further elucidate the connection between different isoforms of bFGF and chemo-resistance, we measured nuclear and cytosolic bFGF levels in chemo-residual TN tumor cells. On western blots, significantly increased levels of nuclear bFGF isoforms (22, 24 kDa), but not the cytosolic bFGF isoform (18 kDa), were detected in chemo-residual cells compared to parental cells (Fig. 2b). This trend was observed regardless of the chemotherapy class studied (doxorubicin or docetaxel, Fig. 2b). By immunofluorescence, we confirmed increased nuclear bFGF in chemo-residual cells relative to parental cells for both SUM159 and BT549 TN breast cancer cells (Fig. 2c) and for two other TN breast cancer cell lines (HS578T and MDA-MB-231, data not shown). These results suggest an association between nuclear bFGF expression and TN breast cancer chemo-resistance.

### bFGF is essential for the survival of chemo-residual tumor cells and subsequent colony formation

**Fig. 3:**
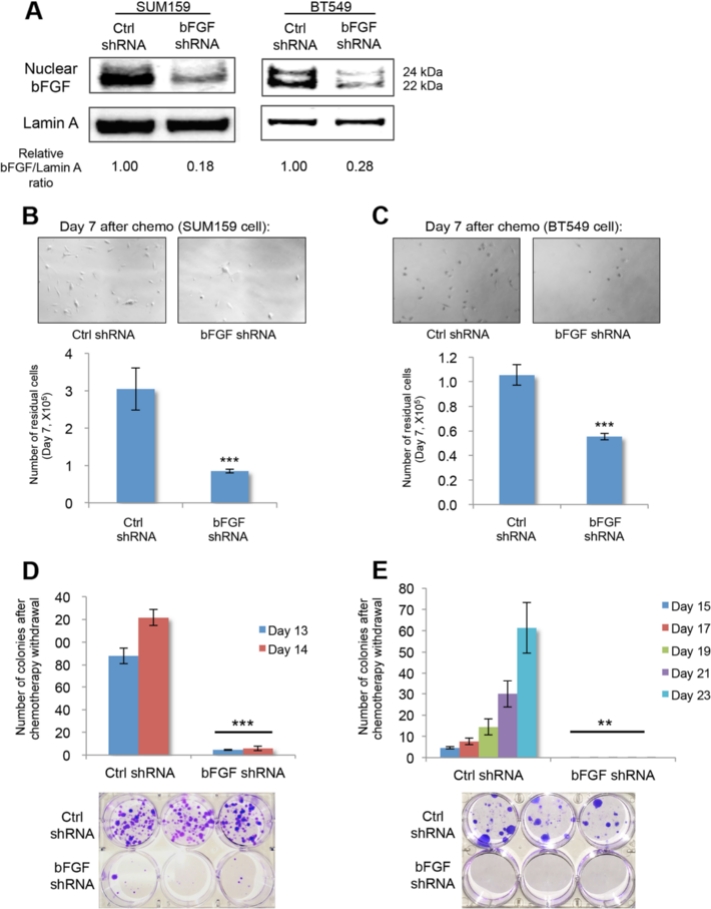
Basic fibroblast growth factor (*bFGF*) knockdown in triple-negative (*TN*) breast tumor cells reduces the number of chemo-residual cells and blocks subsequent colony formation. **a** SUM159 and BT549 cells were transfected stably with a bFGF shRNA or control (*ctrl*) shRNA. The knockdown of nuclear bFGF was confirmed by immunoblotting equivalent amounts of nuclear extract with bFGF antibody. Protein loading was accessed using Lamin A antibody. Protein bands were quantified using Image J software (NIH), and the relative ratio of bFGF to loading control is shown for each lane. **b** and **c** SUM159 cells (**b**) and BT549 cells (**c**) transfected stably with a bFGF shRNA or control shRNA were treated with doxorubicin as described in Fig. 1a. *Upper panel*: pictures of remaining chemo-residual cells were taken on day 7. Magnification ×20. *Lower panel*: numbers of chemotherapy (*chemo*)-enriched chemo-residual cells on day 7 were determined by trypan blue exclusion; n = 3, *error bars* represent SD, ***p <0.001, two-tailed Student’s *t* test. **d** and **e** SUM159 cells (**d**) and BT549 cells (**e**) transfected stably with a bFGF shRNA or control (*ctrl*) shRNA were treated with doxorubicin as described in Fig. 1a. *Upper panel*: colonies (containing >50 cells) were quantified on the indicated days. *Error bars* represent SD, n = 3, **p <0.01, ***p <0.001, two-tailed Student’s *t* test. *Lower panel*: colonies were fixed and stained with crystal violet on day 22 (SUM159 cell) and day 24 (BT549 cell). Similar results were obtained in at least three independent trials

Studies in Fig. 2 indicate that nuclear bFGF is upregulated in chemo-residual TN breast cancer cells. To determine whether bFGF is required for TN breast cancer chemo-resistance, we knocked down bFGF expression in SUM159 and BT549 cells by stable bFGF shRNA transfection (Fig. 3a). Cells transfected with bFGF or control shRNA were treated for 2 days with doxorubicin as in Fig. 1a. The number of doxorubicin-enriched chemo-residual cells on day 7 was significantly decreased in bFGF shRNA transfectants compared to control shRNA transfectants (Fig. 3b, c). Moreover, bFGF shRNA-transfected chemo-residual cells formed dramatically fewer colonies after chemotherapy removal than control shRNA transfectants (Fig. 3d, e). Similar results were observed in two TN breast cancer cell lines (SUM159, BT549; Fig. 3d, e). These results indicate that bFGF is necessary for the survival of chemo-residual tumor cells after doxorubicin challenge and for their subsequent proliferation upon chemotherapy withdrawal.

### Nuclear bFGF promotes the survival of chemo-residual tumor cells and subsequent colony formation

**Fig. 4:**
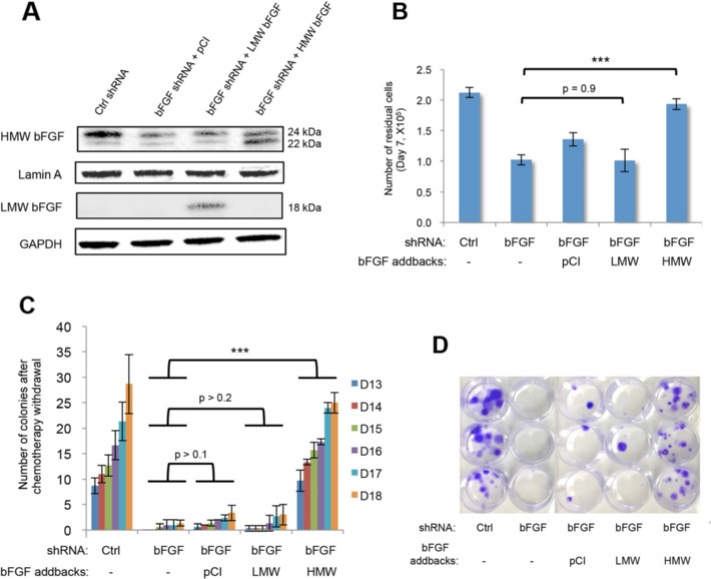
Transfection of basic fibroblast growth factor (*bFGF*) knockdown cells with 23-kDa bFGF (nuclear bFGF) vector restores chemo-residual cell survival and subsequent colony formation. **a** bFGF shRNA-transfected SUM159 cells were transfected with vectors expressing low molecular weight (*LMW*) (cytosolic) bFGF, high molecular weight (*HMW*) (nuclear) bFGF, or pCI as a vector control (*ctrl*). The expression of addback constructs in stable transfectants was confirmed and compared to control shRNA-transfected cells by immunoblotting equivalent amounts of nuclear (*upper two lanes*) or cytosolic (*lower two lanes*) extract with bFGF antibody. Protein loading was assessed using Lamin A or GAPDH antibody. **b** SUM159 cells expressing control shRNA, bFGF shRNA, or bFGF shRNA plus indicated addback constructs were treated with doxorubicin as described in Fig. 1a. The number of chemo-residual cells was determined on day 7 by trypan blue exclusion. *Error bars* represent SD, n = 3, ***p <0.001, two-tailed Student’s *t* test. **c** Colonies (containing >50 cells) were quantified on the indicated days. *Error bars* represent SD, n = 3, ***p <0.001, two-tailed Student’s *t* test. **d** Colonies were fixed and stained with crystal violet on day 20. Similar results were obtained in at least three independent trials

To determine which bFGF isoform facilitates chemo-residual tumor cell survival and colony formation in our model, we transfected bFGF shRNA-expressing cells with a vector expressing 18-kDa rat bFGF, 23-kDa rat bFGF, or an empty control vector (Fig. 4a). The 18-kDa and 23-kDa rat bFGF constructs exhibit 97 % and 82 % homology with human 18-kDa and 24-kDa nuclear bFGF, respectively [30]. The addback of the 23-kDa rat nuclear bFGF, but not the 18-kDa rat cytosolic bFGF, to bFGF shRNA transfectants increased the number of chemo-residual tumor cells to that observed in control cells (Fig. 4b). Likewise addback of the 23-kDa bFGF isoform restored the ability of bFGF shRNA-transfected chemo-residual cells to establish colonies (Fig. 4c, d) following short-term doxorubicin treatment. Of note, similar transfection of SUM159 cells expressing the control shRNA did not influence cell viability (data not shown). Collectively, our results demonstrate that high molecular weight (nuclear) bFGF, but not low molecular weight (cytosolic) bFGF, is sufficient to maintain the viability of chemo-residual tumor cells and promote subsequent colony growth after chemotherapy withdrawal.

### bFGF regulates DNA-PK expression/activity and is associated with accelerated DNA double strand break repair in chemo-residual TN tumor cells

**Fig. 5:**
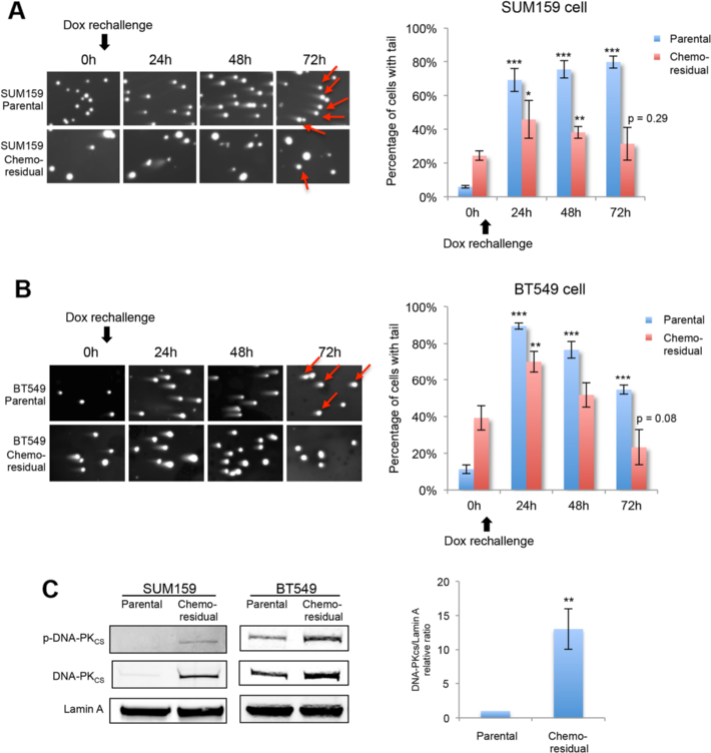
Chemo-residual tumor cells exhibit higher DNA repair capability and increased expression/phosphorylation of DNA-dependent protein kinase (*DNA-PK*_*CS*_). **a** *Left panel*: SUM159 chemo-residual cells and parental (untreated) cells were re-challenged with doxorubicin (*Dox*) (1 μg/ml) for 3 h. Fresh medium was added after chemotherapy removal. DNA damage at sequential time points after chemotherapy treatment was analyzed by neutral comet assay. Representative images are shown at each time point. Cells scored as comet tail-positive are indicated with *red arrows* in the 72-h (*72h*) time frame. *Right panel*: the percentage of cells with comet tails at the indicated time points was quantified with a fluorescence microscope. *Error bars* represent SD, n = 3 fields. Significance of data points at *24h*, *48h* and *72 h* was determined relative to data reported at *0h* for the indicated cell population (*p <0.05, **p <0.01, ***p <0.001, two-tailed Student’s *t* test). Cells scored as comet tail-positive are indicated with *red arrows* in the *72h* time frame. **b.** *Left panel*: BT549 chemo-residual cells and parental (untreated) cells were challenged with Dox (0.5 μg/ml) for 4 h. DNA damage was assessed at the indicated times using the neutral comet assay as in **a**. *Right panel*: the percentage of cells with comet tails at the indicated time points was quantified as in **a**. Cells scored as comet tail-positive are indicated with *red arrows* in the *72h* time frame. **c** *Left* and *middle panel*: SUM159 cells (*left*) and BT549 cells (*middle*) were treated with Dox as described in Fig. 1a. Nuclear protein from parental and chemo-residual cells was extracted. Equivalent amounts were immunoblotted with phospho (Ser 2056)-DNA-PK_CS_, DNA-PK_CS_ or Lamin A antibody. *Right panel*: protein bands from three independent trials (SUM159 cells treated with Dox as described in Fig. 1a) were quantified, and the relative ratio of DNA-PK_CS_ to loading control is shown. *Error bars* represent SD, n = 3, **p <0.01, two-tailed Student’s *t* test

Elevated DNA repair activity is associated with chemo-resistance in many tumors [31–34]. To compare the DNA double-strand break (DSB) repair capability, we re-challenged untreated parental cells and chemo-residual cells with doxorubicin (a DNA-damaging agent) for 3 h and examined their recovery by neutral comet assay. As shown (Fig. 5a, b) the percent cells with comet tails returned to baseline quicker in chemo-residual cells than in parental cells. Similar results were observed in both SUM159 (Fig. 5a) and BT549 (Fig. 5b) chemo-resistance models. These data indicate that chemo-residual TN tumor cells from our short-term chemotherapy treatment model repaired DNA strand breaks more quickly than parental cells.

DNA-PK is the key protein responsible for non-homologous end joining (NHEJ) of DNA DSBs. Over-expression of bFGF in HeLa cells drives the expression and activation of DNA-PK_CS_ [20]. To determine whether DNA-PK_CS_ is a downstream target of nuclear bFGF in our TN breast cancer chemo-resistance model, we determined the expression level of DNA-PK_CS_ in chemotherapy-enriched TN tumor cells. Chemo-residual TN tumor cells expressed increased levels of both DNA-PK_CS_ and phospho (Ser-2056)-DNA-PK_CS_, representing the activated form of DNA-PK_CS_ [35] (Fig. 5c).

### Inhibition of DNA DSB repair by a selective DNA-PK inhibitor decreases the survival of chemo-residual tumor cells and subsequent colony formation

**Fig. 6:**
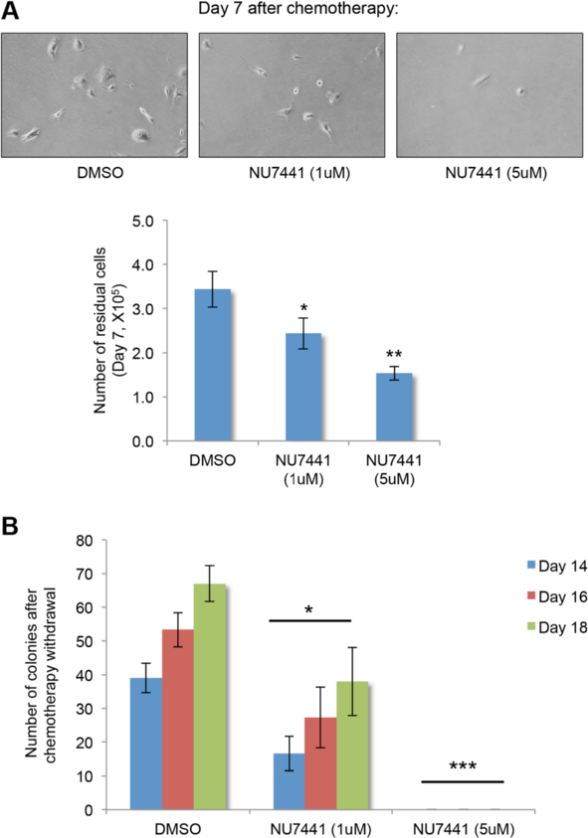
Selective DNA-dependent protein kinase (*DNA-PK*) inhibitor (NU7441) decreases the survival of chemo-residual tumor cells and subsequent colony formation. **a** SUM159 cells were treated with doxorubicin (1 μg/ml) plus dimethyl sulfoxide (*DMSO*) or a selective DNA-PK inhibitor (NU7441), at a non-cytotoxic concentration [36] (1 μM or 5 μM) for 2 days, as described in Fig. 1a. Fresh medium was added after treatment removal. *Upper panel*: pictures of chemo-residual cells were taken on day 7. Magnification ×40. *Lower panel*: Chemo-residual cell number on day 7 was determined by trypan blue exclusion. *Error bars* represent SD, n = 3, *p <0.05, **p <0.01, two-tailed Student’s *t* test. **b** Colonies (containing >50 cells) were quantified on days 14, 16, and 18, respectively. *Error bars* represent SD, n = 3. Significance was determined relative to DMSO-treated cells at each time point using the two-tailed Student’s t test (*p <0.05, ***p <0.001). Similar results were obtained in at least three independent trials

NU7441 is a specific inhibitor of DNA-PK with 100-fold selectivity for DNA-PK, compared to other PI3K kinase family members [36, 37]. To determine whether DNA-PK inhibition reduces TN chemo-residual tumor cell survival and regrowth, we simultaneously treated SUM159 TN breast tumor cells with doxorubicin and NU7441 at either of two non-cytotoxic concentrations [36]. NU7441 significantly decreased the number of chemo-residual cells (Fig. 6a) and subsequent colony formation (Fig. 6b) in a concentration-dependent manner. Previous preclinical studies indicate that the DNA-PK inhibitor NU7441 synergizes with chemotherapy to reduce tumor growth in a colon cancer model [36]. Our results suggest the importance of testing the efficacy of combination therapy (NU7441 + chemotherapy) for TNBC in future preclinical studies.

### Nuclear bFGF drives accelerated DNA repair and DNA-PKcs expression in chemo-residual TN tumor cells

**Fig. 7:**
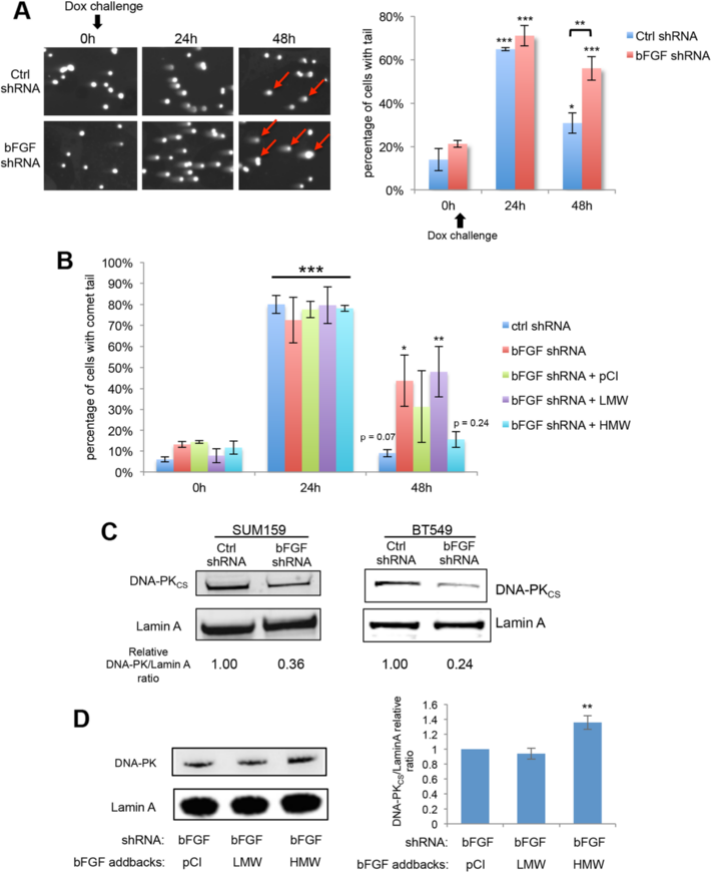
Nuclear basic fibroblast growth factor (*bFGF*) drives DNA repair and DNA-dependent protein kinase catalytic subunit (*DNA-PKcs*) expression. **a** *Left panel*: BT549 cells transfected with bFGF shRNA or control (*ctrl*) shRNA were challenged with doxorubicin (*Dox*) (0.25 μg/ml) for 2 h. Fresh medium was added after chemotherapy removal. DNA damage at sequential time points after chemotherapy treatment was analyzed by neutral comet assay. Representative images are shown for each time point. Cells scored as comet tail-positive are indicated with *red arrows* in the 48-h time frame. *Right panel*: percentage of cells with comet tails at indicated time points was quantified with a fluorescence microscope. *Error bars* represent SD, n = 3 fields (each field containing >50 cells). Significance of data points at 24 and 48 h was determined relative to data reported at 0 h for the indicated cell population (*p <0.05, **p <0.01, ***p <0.001, two-tailed Student’s *t* test). **b** SUM159 cells expressing control shRNA, bFGF shRNA, or bFGF shRNA plus indicated addback constructs (as in Fig. 4a) were challenged with doxorubicin (0.25 μg/ml) for 2 h. Fresh medium was added after chemotherapy removal. DNA damage at sequential time points after chemotherapy treatment was analyzed by neutral comet assay. Percentage of cells with comet tails at the indicated time points was quantified with a fluorescence microscope. *Error bars* represent SD, n = 3 fields (each field containing >50 cells). Significance of data points at 24 and 48 h was determined relative to data reported at 0 h for the indicated cell population (*p <0.05, **p <0.01, ***p <0.001, two-tailed Student’s *t* test). **c** SUM159 and BT549 cells transfected with bFGF shRNA or ctrl shRNA were treated with doxorubicin as in Fig. 1a. Nuclear protein from chemo-residual cells was extracted. Equivalent amounts were immunoblotted with DNA-PK_CS_ and lamin A antibodies. Protein bands were quantified, and the relative ratio of DNA-PK_CS_ to loading control is shown for each lane. **d** *Left panel*: bFGF shRNA-transfected SUM159 cells expressing indicated addback constructs were treated with doxorubicin as in Fig. 1a. Nuclear protein from chemo-residual cells was extracted. Equivalent amounts were immunoblotted with DNA-PK_CS_ and Lamin A antibodies. *Right panel*: protein bands from three independent trials were quantified and the relative ratio of DNA-PK_CS_ to loading control is shown for each line. *Error bars* represent SD, n = 3, **p <0.01, two-tailed Student’s *t* test

We next investigated the effects of bFGF knockdown on DNA repair in chemotherapy-challenged TN breast tumor cells. Twenty-four hours after doxorubicin challenge, SUM159 cells expressing a bFGF shRNA had a similar level of DNA damage to that of cells expressing a control shRNA, with approximately 70 % of cells having a comet tail (Fig. 7a). However, control shRNA-expressing cells exhibited more rapid DNA repair than bFGF shRNA transfectants, with only approximately 30 % of control shRNA-expressing cells having comet tails at 48 h post challenge (compared to approximately 60 % of bFGF shRNA-expressing cells having comet tails at this time) (Fig. 7a). To determine which bFGF isoform drives repair in these cells, we next performed comet assays on knockdown cells reconstituted with low molecular weight (LMW) or high molecular weight (HMW) bFGF. As shown in Fig. 7b, bFGF knockdown cells exhibited significantly slower DNA repair than control cells. Moreover, expression of HMW bFGF, but not LMW bFGF in knockdown cells restored DNA repair to the level observed in control shRNA cells (Fig. 7b).

Based on our observation that DNA repair in chemo-residual cells was associated with increased nuclear bFGF and increased DNA-PKcs levels, we next investigated effects of bFGF knockdown on DNA-PKcs expression. bFGF knockdown significantly decreased the DNA-PK_CS_ protein level in chemotherapy-enriched tumor cells, indicating upstream regulation of DNA-PK_CS_ by bFGF (Fig. 7c). Expression of HMW bFGF, but not LMW bFGF, in bFGF knockdown cells increased DNA-PKcs levels (Fig. 7d). In contrast, expression of HMW bFGF did not influence DNA-PKcs levels in control shRNA cells (data not shown). Collectively, these results suggest that chemo-residual tumor cells support a bFGF/DNA-PK signaling axis that confers accelerated DNA DSB repair capability, allowing them to survive chemotherapy challenge.

### Percentage of nuclear bFGF-expressing tumor cells is increased or maintained in a subset of residual tumors from TN breast cancer patients

**Fig. 8:**
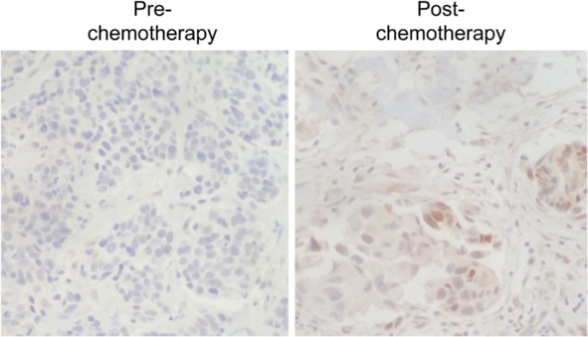
Nuclear basic fibroblast growth factor (*bFGF*) expression is increased in a subset of triple-negative (*TN*) breast cancers post neoadjuvant chemotherapy treatment. bFGF immunohistochemistry was performed on matched tumor tissues obtained from a patient with TN breast cancer before (*pre-chemotherapy*) and after (*post-chemotherapy*) neoadjuvant chemotherapy (docetaxel/cyclophosphamide) treatment. Magnification ×200

To validate our findings in patients with TN breast cancer, we optimized an immunohistochemistry (IHC) protocol for detecting nuclear and cytosolic bFGF in formalin-fixed, paraffin embedded tissues. In a pilot study, we selected nine patients with TN breast cancer that exhibited an incomplete pathologic response to neoadjuvant chemotherapy treatment. Matched samples from patients with TN breast cancer (Fig. 8), obtained before and after neoadjuvant chemotherapy treatment, were stained with bFGF antibody. As shown in Table 1, in five of nine patients, the percentage of nuclear bFGF+ tumor cells increased or remained the same in the post treatment samples compared to those obtained pre-treatment. In contrast, in four of nine patients with TNBC, the percentage of nuclear bFGF+ tumor cells decreased post treatment. These data demonstrate that nuclear bFGF-positive cells are enriched in a subset of patients with TN breast cancer following neoadjuvant chemotherapy treatment. Of note, the percentage of cytosolic bFGF+ tumor cells did not follow the same trends pre-treatment versus post treatment as did the percentage of nuclear bFGF+ tumor cells for these patients (data not shown).

**Table 1:** Nuclear bFGF expression in triple-negative breast tumors before and after neoadjuvant chemotherapy treatment

TNBC patients	Chemotherapy	Nuclear bFGF(+) cells pre-chemotherapy (%)	Nuclear bFGF(+) cells post-chemotherapy (%)	Trend (post-chemotherapy versus pre-chemotherapy)
1	ACT	50	90	Increase
2	TC	1	50	Increase
3	AC	0	100	Increase
4	TAC	100	100	**=**
5	AC	100	100	**=**
6	AC + 1 × paclitaxel	30	20	Decrease
7	ACT	70	20	Decrease
8	ACT	90	25	Decrease
9	AC + 1 × paclitaxel	100	15	Decrease

Nine patients with triple-negative breast cancer (TNBC) exhibiting an incomplete pathologic response to neoadjuvant chemotherapy were identified from medical records under Duke Institutional Review Board approval (protocol 47289). Chemotherapy regimen is indicated (*A* Adriamycin, *C* cyclophosphamide, *T* Docetaxel). Basic fibroblast growth factor (bFGF) expression in formalin-fixed, paraffin-embedded tissues was assessed by immunohistochemistry using bFGF antibody. Nuclear bFGF scoring was performed in a blinded fashion by two pathologists. Consensus scores for percent nuclear bFGF(+) cells are shown. Three of nine patients had increased percent nuclear bFGF(+) cells post-chemotherapy (Increase), two of nine patients had sustained % nuclear bFGF(+) cells post-chemotherapy (=), and four of nine patients had reduced % nuclear bFGF(+) cells post-chemotherapy (Decrease). For the two patients in which % nuclear bFGF(+) cells remained the same post-treatment, 100 % of tumor cells pre-treatment were nuclear bFGF(+). In summary, five of nine cases showed increased or sustained percent nuclear bFGF(+) cells post chemotherapy

## Discussion

Previous chemotherapy selection models have shown the relevance of cancer stem-like cells in TN breast cancer chemo-resistance [27, 28]. In one of these studies, HIF-1α was shown to be a central determinant of TN cancer stem-like cell chemo-resistance [27]. Our model is distinct from these studies in identifying a chemo-resistant TN tumor cell subpopulation enriched after 2 days of chemotherapy treatment followed by a rest period (6 days) during which chemotherapy is removed. We propose that this model is pertinent to the drug holiday experienced in patients between chemotherapy cycles. Notably chemo-resistant cells from our model differ from those arising after continuous chemotherapy treatment. First, chemo-residual TN tumor cells from our model do not exhibit enhanced mammosphere forming ability (Fig. 1c, d), indicating that they do not demonstrate cancer-stem-like cell behaviors. Moreover, HIF-1α, previously implicated in cancer stem-cell chemo-resistance [28], is downregulated in chemo-residual TN tumor cells from our model (Fig. 1e, f). We hypothesize that continuous chemotherapy treatment is necessary to maintain a cancer stem-like cell population, a topic of current investigation in our laboratory. In contrast, our short-term chemotherapy treatment model identifies a chemo-resistant population that is maintained after chemotherapy removal, and that exhibits increased DNA repair capability driven by a nuclear bFGF signaling axis.

Novel markers of TN breast cancer chemo-resistance were recently identified by performing microarray on patient tumor samples obtained before and after neoadjuvant chemotherapy treatment [38]. It is important to note, however, that microarray analyses will not identify chemo-resistance markers regulated at the translational or post-translation level. Our study is unique in studying proteins that are upregulated in chemo-residual TN tumor cells. Using a short-term chemotherapy enrichment model, we have identified a nuclear bFGF isoform, the expression of which is dependent on cap-independent protein translation, that determines survival of TN chemo-residual tumor cells.

Expression of nuclear versus cytosolic bFGF isoforms is determined by alternative translation pathways. Whereas cytosolic bFGF isoforms are regulated by cap-dependent translation, nuclear bFGF isoforms are regulated by cap-independent translation. Notably, we observed increased protein levels of nuclear but not cytosolic bFGF isoforms in chemo-residual tumor cells. Our data suggest that chemo-residual tumor cells may support cap-independent translation, driving expression of nuclear bFGF and DNA repair. We are currently addressing this important hypothesis. Ultimately, it may be possible to eliminate these chemo-residual tumor cells by targeting the cap-independent translation pathway.

Previous studies indicate that tumor resistance to anti-angiogenic therapy is associated with increased expression of cytosolic FGF, which is able to restore tumor angiogenesis [10–15]. Accordingly, we were surprised to observe upregulation of nuclear bFGF isoforms, but not cytosolic bFGF isoforms, in chemo-residual tumor cells. Our studies suggest that nuclear bFGF may drive TN breast cancer resistance in a manner independent of angiogenesis.

We observed that chemo-residual cells, relative to parental SUM159 tumor cells, express increased levels of both the 22-kDa and the 24-kDa nuclear forms of bFGF (Fig. 1d). In order to determine whether both nuclear bFGF isoforms are critical to our model, we performed addback studies using a 23-kDa rat nuclear bFGF construct (exhibiting 82 % homology with human 24-kDa nuclear bFGF). This 23-kDa bFGF isoform alone was able to restore chemo-resistance by promoting chemo-residual cell survival and regrowth in SUM159 cells expressing bFGF shRNA. Our data indicate that the survival function of this 23-kDa bFGF isoform relates to its ability to drive DNA repair.

We also observed that bFGF RNA levels are increased in chemo-residual tumor cells relative to parental tumor cells (Fig. 1c). Identifying signaling pathways that increase bFGF transcription/mRNA stability in chemo-residual cells has the potential to identify rational methods for targeting these chemo-resistant tumor cells. Of note, previous studies indicate that hypoxia drives bFGF transcription in an HIF-1-dependent manner [39]. However, we did not see HIF-1 expression elevated in chemotherapy-enriched tumor cells (Fig. 2e), suggesting an alternative driver of bFGF mRNA expression in chemo-residual tumor cells.

Our work identifies DNA-PK_CS_ as a downstream target of nuclear bFGF in chemo-residual TN tumor cells. DNA-PK_CS_ has previously been implicated in therapy resistance [31–34]. Long term chemotherapy selection models have been shown to select for chemo-resistant tumor cells with increased DNA-PK_CS_ expression/activity [34, 40]. The current study is unique in identifying a nuclear growth factor (nuclear bFGF) in chemo-residual tumor cells that drives DNA repair and DNA-PK_CS_ expression. Notably, nuclear bFGF did not increase DNA-PKcs mRNA levels, as assessed by real-time PCR (data not shown). Further work is needed to determine the mechanism by which nuclear bFGF increases DNA-PKcs expression/activity. In addition, we are investigating the ability of nuclear bFGF to activate other DNA repair pathways.

We also found that a small molecule inhibitor of DNA-PK_CS_ reduced both the number of chemo-residual tumor cells (Fig. 7) and the number of colonies evolving after chemotherapy withdrawal (Fig. 7) in our in vitro model of TN breast cancer chemo-resistance. This activity may be attributed to multiple reported activities of DNA-PK. This DNA-PK inhibitor likely reduces the number of chemo-residual tumor cells by blocking DNA repair, leading to increased tumor cell apoptosis. However, this inhibitor may also chemo-sensitize TN tumor cells by inhibiting a recently reported, non-conventional activity for DNA-PK, namely its ability to induce AKT-dependent cell survival [41]. Finally, DNA-PK is a critical regulator of mitosis [42]. Thus, it is possible that inhibiting DNA-PK in our model prevents the transition of chemo-residual tumor cells to proliferative colonies.

Nuclear-localized EGF receptor is a central determinant of DNA-PK activity [43]. Based on this knowledge, in addition to our current findings, it is intriguing to speculate that nuclear bFGF may control DNA-PK_CS_ expression/activity in a manner dependent on a nuclear bFGF receptor, a topic of current investigation. This possibility is supported by the literature, which demonstrates that nuclear FGF cooperates with a nuclear FGF receptor to drive gene transcription in neurons [44]. Identifying a bFGF receptor that drives nuclear bFGF/DNA-PK signaling has the potential to define a logical therapeutic strategy (i.e., combining chemotherapy with an FGF receptor small molecule inhibitor) to eliminate chemo-residual TN breast cancer cells, thus preventing tumor recurrence.

Our studies of TN breast cancer samples obtained before and after neoadjuvant chemotherapy treatment demonstrate that a subset of these patients exhibit an increased percentage of nuclear bFGF-positive cells post treatment. These data validate our in vitro findings, showing that chemotherapy enriches for nuclear bFGF-positive cells. All of these patients received either anthracycilne or anthracyline + taxane therapy, and exhibited an incomplete pathologic response. While 56 % of matched samples exhibited increased or sustained nuclear bFGF-positive cells, 44 % of matched samples exhibited reduced nuclear bFGF-positive cells post neoadjuvant chemotherapy. Considering that multiple TN breast cancer subtypes have been identified [45], we hypothesize that nuclear bFGF-positive cells may only be enriched in a subset of TN breast cancer subtypes. The role of homologous recombination deficiency (HRD), commonly mediated by loss of BRCA1/2 activity, in chemo-responsiveness is not well-defined, and is actively being investigated (NCT01982448). The HRD/BRCA status of TN breast cancer patients may also influence whether a nuclear bFGF/DNA-PK signaling axis determines chemotherapy resistance. We hypothesize that with follow up the 56 % of patients with increased or sustained percentage of nuclear bFGF-positive cells will exhibit future tumor recurrence. These pilot data underscore the importance of performing a large-scale prospective study of nuclear bFGF expression in TN breast cancer cases before and after neoadjuvant chemotherapy treatment, controlling for BRCA status and TN breast cancer subtype. These follow-up studies have the potential to identify a novel biomarker in a subset of TN breast cancer patients that predicts chemotherapy resistance and future tumor recurrence. In addition, identifying nuclear bFGF as a determinant of chemo-resistance in a subset of TN breast cancers will establish a logical therapeutic strategy for chemo-sensitizing tumors in these patients.

## Conclusions

Using a short-term chemotherapy enrichment model, we demonstrated a critical role for nuclear bFGF in TN breast cancer chemotherapy resistance. Notably, previous continuous chemotherapy selection models did not identify nuclear bFGF as a driver of TN breast cancer chemo-resistance. To begin to demonstrate the relevance of our findings to the clinic, we showed that nuclear bFGF-positive tumor cells were increased or maintained in the majority of patients with TN breast cancer post neoadjuvant chemotherapy treatment. Follow-up studies are needed to determine if nuclear bFGF expression in TN breast cancer cells predicts an incomplete pathologic response and/or future tumor recurrence in patients with TN breast cancer.

Chemotherapy remains the core therapy for TN breast cancer and studies of new agents will likely continue to be done in combination with chemotherapy for the foreseeable future. Our studies suggest that nuclear bFGF maintains survival of a chemo-resistant subpopulation that then drives metastatic recurrence. Developing therapies that target this mechanism may be essential for overcoming this chemo-resistance and reducing TN breast cancer-associated mortality.

## Abbreviations

bFGF: Basic fibroblast growth factor (FGF-2)
DMSO: Dimethyl sulfoxide
DNA-PK: DNA-dependent protein kinase
DNA-PK_CS_: DNA-dependent protein kinase catalytic subunit
DSB: Double-strand break
FBS: Fetal bovine serum
HBSS: Hank's balanced salt solution
HER2: Human epidermal growth factor receptor-2
HIF-1α: Hypoxia inducible factor-1α
HMW: High molecular weight
HRD: Homologous recombination deficiency
LMW: Low molecular weight
PBS: phosphate-buffered saline
RPMI: Roswell Park Memorial Institute
shRNA: Short-hairpin RNA
TN: Triple-negative
TNBC: Triple-negative breast cancer
